# MYC2 signaling in secondary cell wall modulation

**DOI:** 10.3389/fpls.2025.1558922

**Published:** 2025-04-10

**Authors:** Jong Hee Im, Seungmin Son

**Affiliations:** ^1^ Interdisciplinary Graduate Program in Advanced Convergence Technology and Science, Jeju National University, Jeju, Republic of Korea; ^2^ National Institute of Agricultural Sciences, Rural Development Administration, Jeonju, Republic of Korea

**Keywords:** cell wall, jasmonic acid, lignin, MYC2, signaling pathway, stress response

## Introduction

Secondary cell wall (SCW) serves essential biological functions, represents a predominant component of plant biomass, and hold considerable promise for diverse biotechnological and industrial applications. SCW is a highly specialized and intricate structure present in select plant cells, particularly those that necessitate enhanced mechanical strength and rigidity, such as xylem, phloem fibers, and sclerenchyma cells. The molecular composition of the SCW primarily comprises cellulose, hemicellulose, lignin, proteins, and, in certain cases, pectins. These components function synergistically to impart the SCW with its mechanical properties, thereby enabling it to provide structural support to plant cells and facilitating critical processes such as water transport and resistance to mechanical stress. Given the pivotal role of SCW in mediating plant growth, development, and stress tolerance, its modulation needs to be governed by intricate and highly sophisticated regulatory mechanisms. Many studies have demonstrated that SCW biosynthesis is precisely regulated through intricate hierarchical transcriptional networks (Nakano et al., 2015; Zhang et al., 2018a).

More than 20 transcription factors have been identified as key regulators of the sophisticated regulatory cascade controlling SCW formation (Taylor-Teeples et al., 2015). For instance, MYELOBLASTOSIS 46 (MYB46) and MYB83 are acknowledged as pivotal regulators of SCW biosynthesis (Ko et al., 2012). Their promoters are activated by various NAC (NAM, ATAF and CUC) transcription factors implicated in secondary wall regulation, including VASCULAR-RELATED NAC-DOMAIN 6 (VND6), VND7, NAC SECONDARY WALL THICKENING PROMOTING FACTOR 1 (NST1), and SECONDARY WALL-ASSOCIATED NAC DOMAIN PROTEIN 1 (SND1)/NST3 (Zhu and Li, 2021). Subsequently, MYB46 and MYB83 initiate the activation of downstream elements within the SCW transcriptional network, such as MYB58, MYB63, and KNOTTED ARABIDOPSIS THALIANA 7, in addition to cellulose synthase and an array of lignin biosynthetic enzymes. This intricately coordinated regulation facilitates extensive transcriptional reprogramming essential for SCW biosynthesis. Additionally, SCW formation is regulated by the activation and finely tuned regulation of multiple phytohormone signaling pathways, including auxin, cytokinin, brassinosteroid, abscisic acid, and jasmonate (JA) (Didi et al., 2015). Nonetheless, the precise regulatory mechanisms underlying these signaling pathways remain largely unexplored.

**Figure 1:**
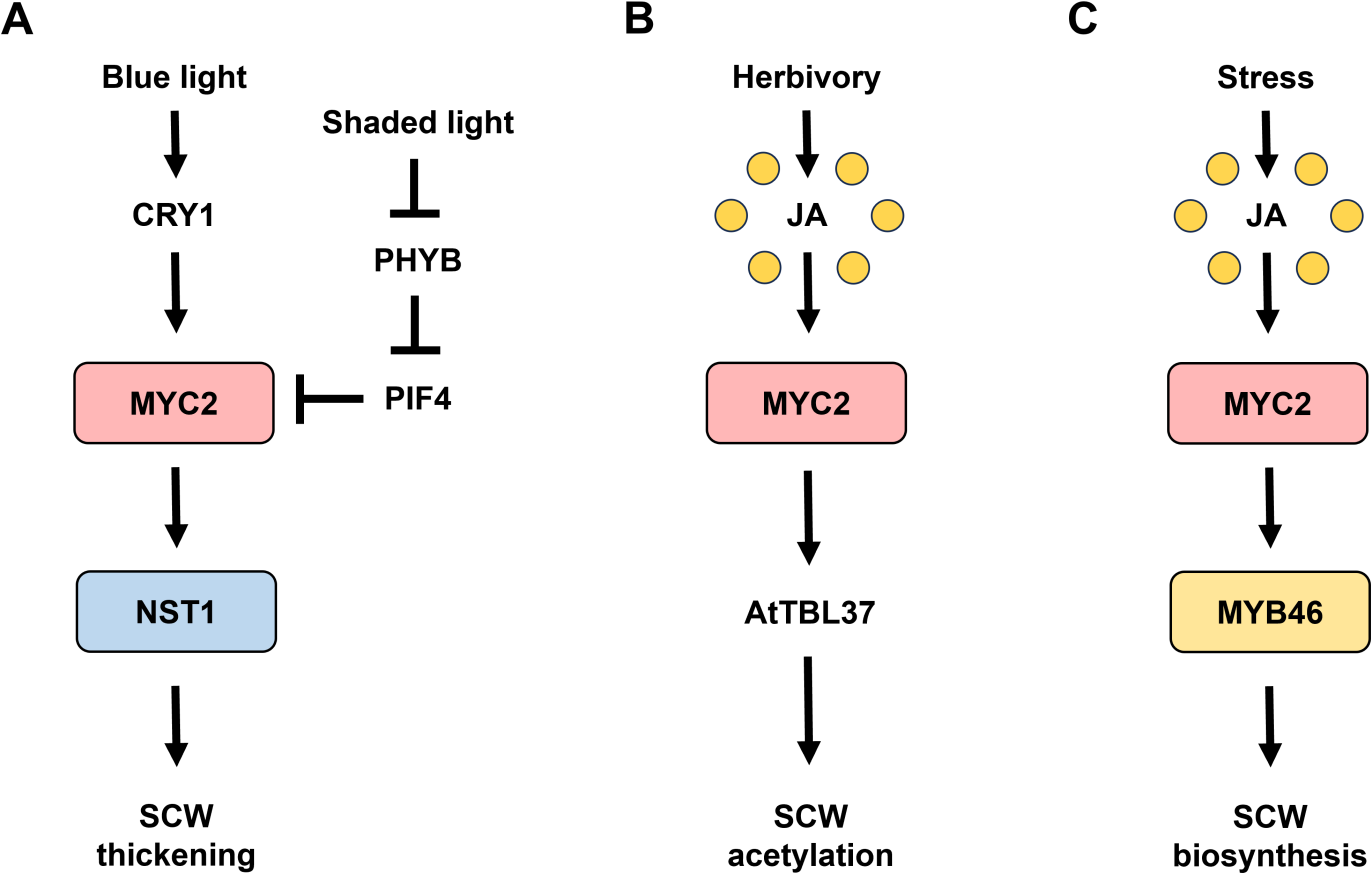
MYC2 signaling in cell wall modulation. **(A)** Light-activated MYC2 signaling in SCW thickening. Blue light perception by CRYPTOCHROME1 (CRY1) activates MYELOCYTOMATOSIS 2 (MYC2), leading to secondary cell wall (SCW) thickening through the upregulation of NAC SECONDARY WALL THICKENING PROMOTING FACTOR 1 (NST1) (Zhang et al., 2018b). Conversely, under shaded light conditions, the inactivation of PHYTOCHROME B (PHYB) results in the nuclear accumulation of PHYTOCHROME INTERACTING FACTOR 4 (PIF4), which promotes the degradation of MYC2, thereby reducing SCW thickness (Luo et al., 2022). **(B)** Herbivory-induced MYC2 signaling in cell wall acetylation. Herbivory-induced damage activates JA signaling, which triggers MYC2 activation. MYC2 then binds to the promoters of *TRICHOME BIREFRINGENCE-LIKE 37* (*AtTBL37*), thereby enhancing cell wall acetylation to strengthen the plant’s defensive response (Sun et al., 2020). **(C)** MYC2-MYB46 module in SCW biosynthesis. Upon activation by JA signaling, MYC2 interacts with and binds to the *MYELOBLASTOSIS 46* (*MYB46*) promoter, thereby facilitating the upregulation of MYB46 expression (Im et al., 2024), contributing to cell wall formation as an adaptive response to environmental stresses.

The basic helix-loop-helix transcription factor (bHLH) MYELOCYTOMATOSIS 2 (MYC2) is master regulator of JA signaling pathway involved in various plant processes including abiotic and biotic stress responses (Song et al., 2022). Recently, the intricate involvement of JA signaling in SCW formation has been determined (**Figure 1**). Considering the critical role of JA-induced MYC2 in mediating SCW formation in response to environmental stimuli, the regulatory mechanisms of MYC2 in SCW modulation require an overview and further discussion.

## Light-activated MYC2 signals in secondary cell wall thickening

Light signaling regulates various plant processes by modulating intricate transcriptional regulatory networks, culminating in extensive transcriptional reprogramming (Jing and Lin, 2020). Varying wavelengths of light influence SCW formation, and some of the detailed molecular mechanisms underlying this process have been elucidated. In *Arabidopsis thaliana* (Arabidopsis), Zhang et al. showed that blue light, mediated by the blue light receptor CRYPTOCHROME1 (CRY1), a key regulator of photomorphogenesis, is involved in modulating SCW formation in the fiber cells of the inflorescence stem, whereas no such effect is observed in the xylem (Zhang et al., 2018b). The blue light signal recognized by CRY1 has the capacity to induce the expression of *MYC2* and its paralog *MYC4*, which function as transcriptional activators by directly binding to the promoter region of *NST1*, thereby initiating a transcriptional cascade that drives the program for SCW thickening; consequently, these findings elucidate a molecular mechanism by which blue light enhances fiber SCW thickening in the inflorescence stems, offering insights into the regulatory interplay between photoreception and cell wall biosynthesis (Zhang et al., 2018b).

Under shaded light conditions, the inactivation of the red to far-red light photoreceptor PHYTOCHROME B (PHYB) results in its cytoplasmic localization, which permits the nuclear accumulation of PHYTOCHROME INTERACTING FACTOR 4 (PIF4) (Luo et al., 2022). PIF4 interacts with the transcription factors MYC2 and MYC4 and promotes their degradation, effectively suppressing their regulatory activity on *NST1* transcription and consequently leading to the inhibition of SCW thickening, thereby unveiling a molecular mechanism through which shaded light negatively regulates SCW thickening in fiber cells of Arabidopsis inflorescence stems (Luo et al., 2022).

## Herbivory-activated MYC2 signal in cell wall acetylation

O-acetylation of wall polysaccharides plays a pivotal role in modulating the structural integrity and biochemical properties of the cell wall, including elasticity, hydrophobicity, and enzymatic interactions, thereby influencing the process of secondary cell wall thickening (Grantham et al., 2017). Furthermore, as cell walls serve as the primary barrier in plant defense against pathogenic invasion, their acetylation status exerts a profound impact on the plant’s ability to mount effective defense responses to pathogens, highlighting the intricate relationship between cell wall composition and plant immunity (Kloth et al., 2019; Munzert and Engelsdorf, 2025). In Arabidopsis, herbivory-induced damage, especially mechanical wounding triggers JA signaling, leading to the activation of MYC2 (Zhang et al., 2017). Sun et al. showed that the activated MYC2 directly binds to the promoters of *TRICHOME BIREFRINGENCE-LIKE 37* (*AtTBL37*)/*SUCROSE-UNCOUPLED 1* (*SUN1*) (Sun et al., 2020). This interaction leads to an increase in the acetylation of cell wall polysaccharides not only in the xylem vessels and interfascicular fiber cells of stems but also in leaf tissues, thereby reinforcing the plant’s herbivore defense through cell wall modification. Moreover, MYC2 and its homologs (i.e., MYC3 and MYC4) play a critical role in modulating cell wall polysaccharide acetylation by regulating the expression of multiple *TBL* genes, thus linking JA-mediated defense pathways to cell wall remodeling (Sun et al., 2020).

## MYC2-MYB46 module in secondary cell wall biosynthesis

Recently, Im et al. uncovered the JA-activated MYC2-MYB46 module as a critical regulator of SCW biosynthesis in the roots and stems of Arabidopsis. JA induces the transcriptional activity of MYB46, and JA-mediated SCW biosynthesis, detected as ectopic lignification, is critically dependent on the function of MYB46, which serves as a key transcriptional regulator of SCW formation (Im et al., 2024). MYC2 has been identified as a direct binding partner of the MYB46 promoter through yeast one-hybrid assays, revealing the regulatory interplay between JA signaling and SCW biosynthetic processes (Im et al., 2024). Notably, MYC2 strongly interacts with the MYB46 promoter, specifically between the −1100 and −900 bp regions upstream of the translation start site, leading to the upregulation of MYB46 expression and subsequent activation of SCW biosynthetic pathways (Im et al., 2024). Diverse genetic evidence has confirmed that MYC2 acts as a direct upstream regulator of MYB46 in response to JA, suggesting that the MYC2-MYB46 module serves as a primary JA signaling hub that modulates SCW biosynthesis in response to various environmental stimuli (Im et al., 2024).

## Perspectives on MYC2-Mediated SCW Modulation

The master regulator of JA signaling, MYC2 plays a central role in SCW formation, with recent studies revealing its function as a regulatory switch influenced by environmental stimulus. The blue light-dependent CRY1-MYC2-NST1 module and the shaded light-responsive PHYB-PIF4-MYC2-NST1 module highlight the complexity of its regulation (Zhang et al., 2018b; Luo et al., 2022), while MYC2-driven pathways, such as MYC2-AtTBL37 for cell wall acetylation and MYC2-MYB46 for SCW biosynthesis (Sun et al., 2020; Im et al., 2024), demonstrate its responsiveness to JA. However, given the interactions of MYC2 with various regulatory factors and its crosstalk with other phytohormones, many underlying mechanisms governing MYC2-mediated SCW biosynthesis remain elusive.

A multitude of studies have reported the importance of lignin deposition and SCW formation in biotic and abiotic stress responses for enhanced resistance (Bachir et al., 2022). Additionally, MYC2 is associated with stress responses, suggesting MYC2 could be a positive regulator of the stresses with SCW regulation. Tailoring MYC2 variants with enhanced stability or refined transcriptional activity presents a promising strategy for optimizing SCW deposition. The development of synthetic promoters responsive to specific light or hormonal cues could enable precise spatiotemporal regulation of *MYC2* expression, while engineered MYC2 fusion proteins may enhance stability or interaction specificity with key SCW regulators. Such targeted modulation of MYC2 holds significant potential for reinforcing SCW biosynthesis in stress-prone environments. Moreover, integrating multi-omics approaches—including transcriptomics, proteomics, and metabolomics—will be essential for unraveling the complex regulatory networks underpinning MYC2-mediated SCW modulation. Therefore, given the pivotal role of MYC2 as a key regulator of SCW formation, future research should focus on deciphering these intricate networks to refine our ability to manipulate SCW biosynthesis, ultimately enhancing plant resilience and optimizing biomass production.
